# Energy-Efficient UAV Movement Control for Fair Communication Coverage: A Deep Reinforcement Learning Approach

**DOI:** 10.3390/s22051919

**Published:** 2022-03-01

**Authors:** Ibrahim A. Nemer, Tarek R. Sheltami, Slim Belhaiza, Ashraf S. Mahmoud

**Affiliations:** 1 Computer Engineering Department, King Fahd University of Petroleum and Minerals, Dhahran 31261, Saudi Arabia; tarek@kfupm.edu.sa (T.R.S.); ashraf@kfupm.edu.sa (A.S.M.); 2 Interdisciplinary Research Center of Smart Mobility and Logistics, King Fahd University of Petroleum and Mineral, Dhahran 31261, Saudi Arabia; slimb@kfupm.edu.sa; 3 Mathematics Department, King Fahd University of Petroleum and Minerals, Dhahran 31261, Saudi Arabia

**Keywords:** UAV, fairness, coverage score, reinforcement learning, actor–critic

## Abstract

Unmanned Aerial Vehicles (UAVs) are considered an important element in wireless communication networks due to their agility, mobility, and ability to be deployed as mobile base stations (BSs) in the network to improve the communication quality and coverage area. UAVs can be used to provide communication services for ground users in different scenarios, such as transportation systems, disaster situations, emergency cases, and surveillance. However, covering a specific area under a dynamic environment for a long time using UAV technology is quite challenging due to its limited energy resources, short communication range, and flying regulations and rules. Hence, a distributed solution is needed to overcome these limitations and to handle the interactions among UAVs, which leads to a large state space. In this paper, we introduced a novel distributed control solution to place a group of UAVs in the candidate area in order to improve the coverage score with minimum energy consumption and a high fairness value. The new algorithm is called the state-based game with actor–critic (SBG-AC). To simplify the complex interactions in the problem, we model SBG-AC using a state-based potential game. Then, we merge SBG-AC with an actor–critic algorithm to assure the convergence of the model, to control each UAV in a distributed way, and to have learning capabilities in case of dynamic environments. Simulation results show that the SBG-AC outperforms the distributed DRL and the DRL-EC3 in terms of fairness, coverage score, and energy consumption.

## 1. Introduction

Improvement in UAV-based methods and the expense decrease of their sensing devices have offered a great foundation for wireless technologies. Due to the characteristics of having a small volume, high three-dimensional (3D) mobility, low energy consumption, and a higher chance of line-of-sight (LoS) [1], UAVs have been widely used in emergency rescue, civil, public, and military applications [2,3,4]. In various applications, with proper operation and placement, UAVs can be deployed either as extra access points to enhance the communication performance of the network or relays to disseminate data. Furthermore, UAVs can work as self-organizing nodes and can efficiently process the preassigned tasks [5]. Hence, a UAV-based network can provide efficient and reliable wireless communication approaches for different real-time scenarios such as public safety scenarios. Here, the UAVs can be deployed as flying base stations (BSs) to replace the defective communication network and guarantee the transmission of data for the ground users [6].

Despite the promising future and the numerous dazzling potentials for using UAVs as flying BSs, different technical issues usually appear in the UAV networks, and these should be avoided and minimized to have a reliable and efficient network. The most important issues include area coverage, 3D deployment/trajectory, communication connectivity, resource allocation, and energy limitations [4,7,8,9,10]. In particular, the deployment of UAVs for coverage and tracking purposes highly impacts the energy resources, the connectivity among the UAVs, the limited communication range, and the interference produced by UAVs. Hence, it is difficult for UAVs to cover the candidate region all the time. Therefore, UAVs need to move around to ensure each ground point is covered for a specific duration. In the UAV area coverage problem, the important part is the evaluation of the coverage ability for the model. Another important factor in the coverage problem is the fairness; the UAVs should cover all parts of the region rather than covering only certain areas and leaving others without coverage [11,12].

UAV-communication-based research can be divided into two main categories: one is extending the communication coverage of the UAV network and deploying the UAVs as mobile BSs to serve the ground points, as in [13,14,15]; the second category is relay UAVs, where UAVs are used to forward data from one point to the next UAV or to the ground point with minimum resources, as in [16,17,18]. In addition to the coverage problem, energy is another issue for the UAV network since UAVs are generally powered by limited batteries. With limited energy resources, UAVs cannot keep moving all the time, and this leads to performance and endurance degradation; hence, UAVs need to work in an energy-efficient way to increase the network lifetime, keep the UAVs connected, and utilize the UAVs’ resources. Given that the UAV coverage and energy control issues are even more complex and challenging than other traditional control issues, a decision-making technique is needed to manage the interactions among multiple UAVs to achieve multiple objectives at the same time.

One of the decision techniques that is widely used to study the coverage and energy problems in UAV networks is game theory (GT). It is a powerful tool for mathematically modeling the interaction among the UAVs in the network to establish the coupling relationships of various rational decision-makers and to achieve an efficient distributed management of the network. Moreover, it can be used to design the distributed control strategies in the UAV network, and it has the ability to find the optimal values of the control strategies [19]. Different types of GTs have been used to study the interactions among the UAVs and solve the coverage and energy issues in UAV networks such as the coalition formation game [20], potential games [21], and the mean field game (MFG) [22].

Another type of solution that can be used to solve the energy and coverage problem is by leveraging deep reinforcement learning (DRL). It has shown a superior performance compared with the optimization- and game-based approaches [23,24,25,26]. The basic DRL algorithm, deep Q learning, depends on the deep Q network (DQN) to find the Q-value for each action–state pair, but it has a limited action space. Due to the unlimited action space for the coverage and energy control problem in the UAV network, a deep deterministic policy gradient (DDPG) method can be used instead of the basic DRL [27,28]. In this research, the control problem is complex since it needs to optimize four objectives at the same time: coverage ability, energy consumption, connectivity, and fairness. Therefore, the DDPG is a promising solution, and it can be used along with the designed utility of the game model to achieve more coverage, less energy consumption, and high fairness, while keeping the UAVs connected all the time [29,30,31]. It can also deal with complex state spaces and with time-varying environments, and it uses powerful deep neural networks (DNNs) to assist the UAV in making decisions and providing high-quality services for the UAV network. Moreover, the DDPG has the ability to deal with unknown environments and emergency scenarios, and it enhances the robustness and reduces the calculation cost of the UAVs.

UAVs with high mobility properties need to work in a team to provide an effective communication coverage for a long period. This mission is challenging since UAVs have in general a limited energy resource and communication range. The first concern is that it is difficult to have the appropriate UAV coverage of the candidate region for a long period, due to the costs and the limitations in the communication range. Indeed, UAVs sometimes need to fly around to guarantee that the region is being covered during the required period. It is also important to have a fair communication within the region, as it is not efficient to cover part of the region for the whole period and leave some other parts without coverage. The second concern is the limitation in the energy resources as UAVs do not have the ability to keep flying for a long period. Hence, they need to use their energy resources in an efficient way in order to increase their lifetime. UAVs’ movements should also be optimized in order to complete more tasks with minimum energy requirements. In addition, movement control for a number of UAVs is quite challenging due to the huge number of possible interactions among UAVs.

To address the coverage and energy challenges, considering the limitations of the existing models such as working in a dynamic environment, complexity, and high computational time, we need to propose a solution approach able to achieve a fair communication and maximize the covered region of the UAVs with minimum energy resources. For this purpose, we propose a game theoretic model with online learning capabilities. This model is able to update the strategies following the environment dynamics and the mobility issues. The main contributions of this paper are as follows:

- We modeled energy-efficient UAVs’ controls, which provide a fair communication coverage for all ground cells in the candidate region;
- We developed a distributed UAV deployment algorithm using GT, which simplifies the interactions among UAVs and provides an optimal solution for the proposed game;
- We propose a learning model that handles cases such as time-varying environments and sophisticated state spaces and to guide the decision-making process.

The remainder of the paper is organized as follows. Section 2 reviews the available game- and learning-based models. Then, the system model and the problem definition are presented in Section 3. Section 4 formulates the state-based game model and presents the detailed design of the proposed DRL model. The simulation setting and the performance evaluation are provided in Section 5. Section 6 concludes the paper.

## 2. Related Works

In this section, we review the recent game- and learning-based research, especially those that studied the deployment, coverage, and energy problems in UAV-based networks. We divide this section into game-based models and learning-based models, and we then summarize the most important models as follows.

### 2.1. Game-Based Models

In [20], Ruan et al. presented an efficient cooperative UAV deployment method and analyzed data transmission and UAV coverage in the UAV-assisted network. They designed it based on a coalition formation game (CFG) with Pareto order. Then, they combined the game model with the coverage deployment and coalition selection approach, so the UAVs can select their strategies in a cooperative manner to improve the coverage ability. Furthermore, Ruan et al. in [21] proposed a multi-UAV coverage model with energy-efficient communication. It consisted of the coverage maximization problem and the power control problem. The coverage algorithm was designed using a spatial adaptive gameto maximize the coverage with minimum transmission power.

For cooperative search and surveillance purposes, Li and Duan in [32] presented a game theoretic model for a multi-UAV network. They divided the cooperative search problem into coordinated movements, sensor observations, and cooperative data fusion. The coordinated movements task was studied as a multi-player potential game, and then, they used a binary log-linear learning algorithm to perform and control the movements of the UAVs in a way that would achieve the optimal coverage. Next, they used a cooperative data fusion algorithm to build the probability map and, hence, guide the next coordinated movements. In [33], Xing et al. proposed a distributed algorithm with cooperative and reliable data transmission for a dynamic environment. To investigate the interactions among UAVs, a game framework was presented with a utility function that included the delay, achievable rate, and energy consumption of the UAVs. They established a multi-hop tree structure network between the UAVs and the BS using a hybrid network formation algorithm.

Another type of game called MFG was used in [34]. Gao et al. formulated the velocity control problem as the Schrödinger bridge problem for a set of massive rotary wing UAVs. It helps describe the location dynamics of the UAVs and their frequent reconfiguration. To reduce the computational complexity and achieve a stable and rapid coverage approach, they transformed it into an MFG and then solved it using the Gprox primal dual hybrid gradient (PDHG) technique.

### 2.2. Learning-Based Models

Li et al. in [35] used a non-cooperative game concept with a modified binary log-linear technique in order to achieve fast and efficient deployment, while searching for an optimal Nash equilibrium point. The technique not only considers the power management and channel overlap, but also the interference and coverage problem. They used an aggregative game that catches and covers all features to overcome the limitations in post-disaster situations. In order to lessen the exchange of information and minimize computational time, they introduced a synchronous payoff-based binary log-linear learning approach. Another research work focused on minimizing the energy consumption [29]. Liu et al. proposed a cooperative approach based on an actor–critic algorithm to provide coverage for unknown areas while reducing the overlap of the UAVs’ views. Game theory was used to solve the complex dynamics of the UAVs. Moreover, a gradient approach was designed to deal with the large state space by reducing the required space to store variables.

Another set of research works studied the coverage and fairness problems [22,31,36,37]. Liu et al. in [31] presented a DRL-based energy-efficient control for coverage and connectivity (DRL-EC3), and it was an energy-efficient UAV control approach based on deep reinforcement learning (DRL) technology. DRL-EC3 explicitly uses a new energy-efficient mechanism based on the deep deterministic policy gradient (DDPG), while taking into consideration the energy consumption, communication coverage, fairness, and connectivity among UAVs. It takes actions based on the learning of the two DNNs for the actor and critic networks. The simulation results showed that DRL-EC3 outperformed random and greedy in coverage, consumption energy, fairness, and energy efficiency. Furthermore, in [36], Liu et al. suggested a distributed deep reinforcement learning approach for controlling the UAV in a decentralized way. The approach increases the average coverage score of the UAVs, improves the fairness of all targeted points, and decreases the overall energy consumption while achieving higher connectivity among UAVs and keeping them flying inside the targeted region. They specifically designed each UAV using DNNs according to the action space, reward, observation, and state in a straightforward way.

In [22], Chen et al. modeled the UAV control problem using an MFG to simplify the interactions among UAVs. The mean-field trust region policy optimization (MFTRPO) uses the MFG to build the Hamilton–Jacobi–Bellman and Fokker–Planck–Kolmogorov equations. It uses neural network feature and trust region policy optimization to solve the difficulties in practical applications and to reach the optimal solution. It improves the communication efficiency while guaranteeing network connectivity and fair communication. Pham et al. in [37] presented an algorithm for multiple agents based on reinforcement learning, where UAVs can collaborate and learn to cover an unknown field of interest, while reducing the resulting overlap of the UAVs’ views. The complexities of the UAV team’s joint actions were resolved by designing a game approach. The fixed sparse representation (FSR) and radial basis function (RBF) techniques were also used to solve the problem in a high-dimensional state space, thus significantly reducing the space to store the parameters.

Researchers have also investigated the power control problem [38,39]. In [38], Yuan et al. discussed the backhaul power allocation and user-timeslot scheduling problem to reduce the energy consumption of UAVs. They proposed two learning models based on actor–critic deep reinforcement learning called joint actor–critic-based user group scheduling and optimization-based backhaul power allocation (ACGOP), and actor–critic-based user group scheduling and backhaul power allocation (ACGP). ACGOP combines optimization and the actor–critic (AC) to speed up and improve the learning performance. Furthermore, they developed reward re-design and action filtering approaches to decrease the large action space and ensure feasibility. Li et al. in [39] studied the power control problem in an ultra-dense UAV network to improve the energy efficiency (EE). They formulated the downlink power control problem as a discrete MFG to simplify the interactions among the UAVs. Then, a Markov decision process (MDP) was constructed using the MFG framework due to the dense deployment of the UAVs to find the equilibrium point. Furthermore, they extended the game by DRL-MFG using the DRL technology to reduce the interference and improve the EE in the network.

The capacity, coverage, and energy efficiency problems were studied in [40,41]. In [40], Atli et al. developed a Q-learning-based UAV placement strategy to solve the coverage and capacity needs in terms of transmit power, altitude regulations, and non-flight zones for long-term wireless communication. They focused on finding the best location for the UAV-BS that would reduce the energy consumption and increase the coverage score. The weighting method in the suggested Q-learning-based solution allows prioritizing the coverage score and energy usage based on the network/battery circumstances. Furthermore, it uses the standard k-means clustering method to place the UAV-BS at the centroid location with the minimum distance to the ground users. Zhang et al. in [41] presented a DRL-based self-remedy approach called SREC-DRL to improve the user satisfaction scores for a specific time period when at least one UAV exits the UAV network. They trained the DDPG agent to proactively relocate UAVs in the network when one UAV was about to quit rather than to start the relocation process after one UAV quits.

Another set of research works focused on the trajectory design for UAV networks [42,43,44,45]. Cui et al. in [42] proposed a DDPG algorithm for power allocation and 2D UAV trajectory design to maximize the downlink throughput and UAV’s service time with minimum energy resources. In [43], Zhang et al. studied the trajectory design of the multi-UAV network to achieve better downlink capacity in the communication system under the coverage constraint. The 3D movement of UAVs under the coverage constrain was modeled as a constrained Markov decision process (CMDP) problem. To solve this problem, they used a constrained deep Q-network (cDQN) algorithm, where each UAV serves as an agent to search and learn its 3D movement policy. The purpose of the cDQN was to maximize the capacity and ensure that all ground points were covered in the system. They also used a primal-dual method in the training of the primal and dual variables, and they applied action filtering to remove the wrong actions.

In [44], Ding et al. presented the 3D trajectory and frequency allocation problem in terms of the fairness and energy consumption of the UAV network. They formulated the energy consumption equations of the UAVs as a function of the 3D locations and defined the fair throughput in a way that would be improved with limited energy. Hence, they proposed a deep reinforcement learning (DRL) approach called energy-efficient fair communication through trajectory design and band allocation (EEFC-TDBA). It was designed to maximize the fair throughput with the minimum energy resources. In [45], Qin et al. discussed the trajectory optimization problem of multiple UAV-BSs in a dynamic environment for user-fair communication service. They characterized the user fairness by using the fairness scheduling, and then, they formulated a weighted throughput maximization problem as a function of the UAV-BSs’ trajectory. They also modeled the dynamic deployment problem as a Markov game with multi-agent DRL-based distributed UAV-BS control called MAUC. It adopts the centralized training framework with the distributed execution.

We found from the previous works and the summarized features in Table 1 that GT allows mathematically modeling the interactions among UAVs and constructing the appropriate order relationships for the different decision-makers. GT is widely applied in the formulation of wireless communication issues, such as coverage, fairness, and power control problems. Potential games are suitable for modeling the coverage and energy problems with medium and small UAV networks [20,21,32]. On the other hand, MFG in [34] was used for modeling the energy efficiency in large UAV networks due to its ability to handle huge interactions among UAVs. In some scenarios, UAVs might be distributed in unknown and dynamic environments; hence, the game models alone will not achieve an optimal solution for the UAV network, Therefore, UAVs should have some learning capabilities such as the DRL, DQN, and actor–critic algorithm (DDPG) as in [22,31,36,37]. These learning techniques can be used for complex state spaces and time-varying environments. Furthermore, it consists of DNNs that serve to decide the suitable actions for the UAVs and achieve the convergence and robustness of the network.

## 3. System Model

This section details the system model components (scenario, channel, energy consumption), states the problem, and discusses the solution approach in order to improve the network performance.

### 3.1. Scenario

In this paper, we deployed a number of *N* UAVs (U) from a number of aerial base stations. The UAVs were equipped with GPS and had the ability to fly within a limited altitude level in order to provide a fair coverage to all cells in the candidate region. The UAVs $\;U_{1},\;U_{2},\;\ldots ,\;U_{N}$ were aware of their own locations. The transmission powers of the UAVs are defined as $P_{u_{1}},\;P_{u_{2}},\;\ldots ,P_{u_{N}}$. They had the ability to change their 3D locations and track the on-ground users. This provides a better coverage and a higher wireless service quality with minimum energy requirements. Each UAV had connectivity limitations, such as a communication/sensing range $R_{c}$. The UAV would lose its links to the other UAVs when the communication range was less than the separating distance. The UAVs also had maximum flying altitude values. Therefore, their coverage range was limited by their physical specifications and the environment conditions.

**Figure 1:**
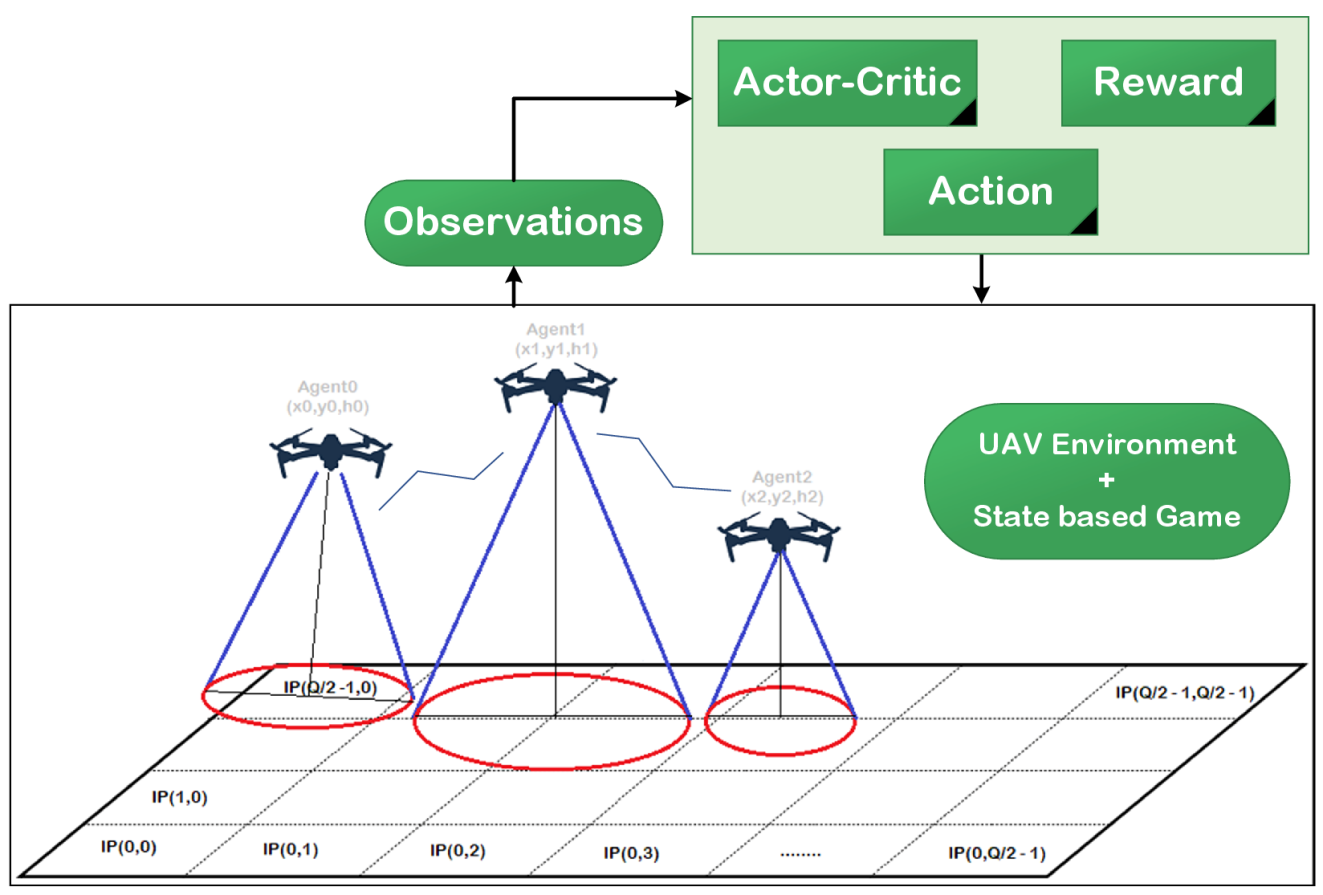
UAV network with the actor–critic algorithm.

To simplify the coverage and energy issues and facilitate the representation of the UAV-based network, the candidate region $\left(R\in R^{2}\right)$ was divided into *Q* cells, and the center of each cell is referred to as the point of interest (IP), as illustrated in Figure 1. Each UAV was required to cover a number of IPs in a reasonable time based on the mission. The communication process can be achieved in *T* slots of equal duration, where each slot is denoted ΔT. For the sake of simplicity, we assumed that the signal over the cell could be wholly specified by the UAV when its center was within the sensing range. To clarify the “points of interestor IPs” term, we assumed that there was a user in the center of this cell (IP). Due to the limited number of UAVs and the energy and coverage challenges, UAVs cannot always guarantee the coverage of all IPs in the region. Moreover, the distribution of UAVs in practical scenarios is considered as a random and independent process, since UAVs are generally deployed in an unplanned and opportunistic manner. At the beginning of the mission, the UAVs started from random locations, and in each timeslot, the UAV could hover or move to the next location based on the 3D space, as explained later in the game model.

Let $l_{u}\left(t\right)={\left[x_{u}\left(t\right),\;y_{u}\left(t\right),\;h_{u}\left(t\right)\right]}^{T}$ represent the 3D location of a given UAV at time *t*, where $\left(x_{u}\left(t\right),\;y_{u}\left(t\right)\right)$, and $h_{u}\left(t\right)$ are, respectively, the UAV’s coordinates in the ground-level horizontal plane and the UAV’s altitude at time *t*. Due to the limited flight speed of the UAV, its trajectories are defined by the maximum moving distance and can be expressed as follows:

(1) $$∥l_{u}\left(t+1\right)-l_{u}\left(t\right)∥\leq v_{u}T_{flight},$$

where $v_{u}$ represents the flight speed of the UAV ${(U}_{u})$ and $t_{flight}$ is the time needed to travel from the start location to the destination location. An additional constraint was introduced to avoid collisions that could occur between any two UAVs $\left(U_{i},U_{j}\right)$. It is expressed as follows:

(2) $$∥l_{i}\left(t\right)-l_{j}\left(t\right)∥\geq d_{min},$$

where i,j∈{1,…,N}, i≠j, and $d_{min}$ represents the minimum distance to keep between the two UAVs (i,j) to avoid collision and interference issues.

### 3.2. Channel and Coverage Model

We assumed that the link between any IP k∈{1,…,Q} and UAV u∈{1,…,N} follows the line-of-sight (LoS) and non-line-of-sight (NLoS) propagation models. As detailed in [13], a channel in a free space path-loss model can be expressed as follows:

(3) $$\xi_{dB}=10\times n_{0}\times \log\left(\frac{4\pi f_{c}d_{uk}}{c}\right),$$

where $f_{c}$ is the system carrier frequency, $d_{uk}$ represents the distance between the UAV and IP, *c* is the speed of light, and $n_{0}$ is the path-loss exponent specific to the environment (i.e., rural, urban, dense urban), as in [46]. A popular approach used to model the UAV-to-ground (U2G) links is the probabilistic LoS and NLoS [13], where the NLoS results usually from the shadowing and diffraction issues in the environment, and the resulting attenuations in the NLoS have a greater effect on the UAVs compared to the LoS. As in [47], the path-loss between UAV *u* and IP *k* is expressed as follows:

(4) $$L_{uk}\left(dB\right)=\left\{\begin{array}{c} \xi_{uk}+\chi_{LoS},\;\;\;\;LoS\;Link.\\ \;\;\;\;\;\xi_{uk}+\chi_{NLoS},\;\;\;\;NLoS\;Link. \end{array}\right.$$

where $\chi_{LoS}$ and $\chi_{NLoS}$ represent the additional attenuation caused by the shadowing problem. In this system, the probability of the LoS link depends on a set of variables based on the environment such as the IP’s and UAV’s locations and the elevation angle between the IP and UAV. Therefore, the LoS and NLoS probabilities can be expressed [48] as follows:

(5) $$P_{uk}^{LoS}\left(t\right)=C{\times \left(\frac{180}{\pi }\times \theta_{k}\left(t\right)\right)}^{B},$$

(6) $$P_{uk}^{NLoS}\left(t\right)=1-P_{uk}^{LoS}\left(t\right),$$

where *C* and *B* are constant parameters that depend on the environment, and the elevation angle θ can be obtained as follows:

(7) $$\theta \left(t\right)={sin}^{-1}\left(\frac{h_{u}-h_{k}}{d_{uk}\left(t\right)}\right)\;,$$

Here, $h_{u}$ and $h_{k}$ represent the altitude of the UAV and the altitude of IP *k* from the ground level, respectively. The horizontal coordinates of point *k* and the horizontal distance from the UAV at time *t* are expressed by $\left(x_{k},y_{k}\right)$ and $r_{uk}\left(t\right)=\sqrt{{\left(x_{u}\left(t\right)-x_{k}\right)}^{2}+{\left(y_{u}\left(t\right)-y_{k}\right)}^{2}}$, respectively. Let $\;d_{uk}\left(t\right)=\sqrt{{\left(r_{uk}\left(t\right)\right)}^{2}+{\left(h_{u}\left(t\right)-h_{k}\right)}^{2}}$ represent the 3D distance between the UAV and IP *k* at time *t*. As in [13,43], the average path-loss can be expressed as follows:

(8) $${\bar{L}}_{uk}\left(t\right)=P_{uk}^{LoS}\left(t\right)\times L_{uk}^{LoS}+P_{uk}^{NLoS}\left(t\right)\times L_{uk}^{NLoS}.$$

The coverage probability of the IP can be evaluated by using the average path-loss between the IP and the UAV. When the IP falls within the communication range of the UAV, we considered that this IP was covered. We also assumed that any given IP can be covered by many UAVs at the same time. As in [49], the corresponding coverage value of any cell $\left({IP}_{i}:\;i\in \left\{1,\ldots ,Q\right\}\right)$ at time *t* was considered as a control strategy for the network and can be expressed as follows:

(9) $${IP}_{i}\left(t\right)=\left(1-\prod_{\;j\in N}\left(1-P_{cov\left(i,j\right)}\left(t\right)\right)\right).$$

The main objective of the study was to maximize the overall coverage score with minimum energy requirements. However, in such cases, this might cause unfair coverage for some IPs in the candidate region. In other words, some IPs could be covered for a long time, while other IPs could be rarely covered during the mission period. Therefore, we needed to guarantee a fair coverage for all IPs in the region. This can be carried out by a measurement metric called Jain’s fairness index (FI) [50]. By adopting the predefined control strategy, the corresponding FI value is expressed as follows:

(10) $$FI=\frac{{\left(\sum_{i=1}^{Q}{IP}_{i}\right)}^{2}}{Q\left(\sum_{i=1}^{Q}{{IP}_{i}}^{2}\right)}.$$

### 3.3. Energy Consumption Model

In general, the UAVs’ energy consumption model consists of two main parts, namely: the movement/propulsion part and the communication part. The movement/propulsion power consists of three parts: induced power, profile power, and parasitic power. The induced power results in the thrust of the propelled air downward. The power of the profile overcomes the rotational drag experienced by the spinning blades of the propeller. The parasitic force avoids body drag when there is a relative translational displacement between the quadrotor and the wind. Based on the previous description, the movement/propulsion energy of the UAV is employed to provide thrust to overcome gravity and the drag during the movements. The flight movements of a UAV can only be horizontal movement, hovering, and vertical movement. Some factors affect the power consumption model such as payloads, the weight of the UAV, and flight time [51]. On the other hand, the energy for communication results from signal radiation/reception, signal processing, and the communication circuitry. Specifically, this consumed energy is often smaller compared with the flight energy [52]. Therefore, the energy for communication was neglected in this research. Hence, the approximated movement/propulsion power can be expressed mathematically as follows [53,54]:

(11) $$P_{u}\left(v_{u}\right)=P_{0}\left(1+\frac{3v_{u}^{2}}{U_{tip}}\right)+P_{1}\sqrt{\left(\sqrt{1+\frac{v_{u}^{4}}{4v_{0}^{4}}}-\frac{v_{u}^{2}}{{2v}_{0}^{2}}\right)}+\frac{d_{0}\rho s_{0}Av_{u}^{3}}{2},$$

where $U_{tip}$ is the tip speed for the rotor blade on the UAV, $d_{0}$ represents the fuselage drag ratio for each rotor, ρ is the air density, $s_{0}$ represents the rotor solidity, *A* is the disc area for each rotor, $v_{0}$ is the mean rotor-induced velocity in the hovering mode, and $P_{0}$ and $P_{1}$ are the blade profile power and the derived power, respectively.

Hence, the energy consumption can be written as follows:

(12) $$E_{\mathrm{cons}}\left(t\right)=P_{u}\left(v_{u}\right)T_{flight,u}\left(t\right)+P_{u}\left(0\right)\left(\Delta T-T_{flight,u}\left(t\right)\right),$$

where $T_{flight,u}\left(t\right)=\frac{∥l_{u}\left(t\right)-l_{u}\left(t-1\right)∥}{v_{u}}$, and it represents the flight time for the UAV (u) in timeslot *t*.

Furthermore, the residual energy $\left(E_{\mathrm{res}}\right)$ in timeslot (t) can be defined in terms of the consumption energy ${(E}_{\mathrm{cons}})$ and the battery size $\left(E_{\max}\right)$ as follows:

(13) $$E_{\mathrm{res}}\left(t\right)=E_{\max}-E_{\mathrm{cons}}\left(t\right).$$

Once the UAV reaches a minimum energy called $E_{\min}$, it quits from the system, and it goes to recharge.

### 3.4. Problem Statement

UAVs should perform reasonable movements in order to provide a fair and effective communication coverage to all IPs. However, to maintain the connections among the UAVs as much as possible and reduce the energy consumption, we should reduce the movements of the UAVs. In short, our target was to find a control algorithm that can meet the following objectives:

1. Maximize the total coverage score in the network;
2. Maximize the geographical fairness to provide a fair and effective communication to all IPs;
3. Minimize the energy consumption resulting from the movements of the UAVs;
4. Ensure the connectivity between the UAVs in the network, avoid crossing the borders of the candidate region, avoid collisions between the UAVs, and optimize the UAVs’ movements in the network;
5. The UAV network should have online learning capabilities, especially in the case of unknown environments or sudden changes in the candidate region during a mission.

## 4. Problem Formulation

In this section, we develop a state-based potential game to move the UAVs from random initial locations to appropriate locations in order to achieve maximum coverage with minimum energy consumption. Then, we introduce a learning algorithm to update the UAVs’ actions until reaching a steady-state point without any improvement.

### 4.1. Game Formulation

The main idea was to propose a distributed algorithm that can implement and simplify the interactions among the UAVs based on GT. In general, GT is adopted and used in most of the previous UAV-based research to analyze interactions among UAVs, especially in dynamic and dense networks. UAVs in the game approach are considered as the players of the game and can interact to make decisions based on the available strategies. They can play in a smart manner by selecting the best strategy that maximizes the coverage score and achieves fair communication coverage with minimum energy consumption. Our game consisted of three main components:

1. Set of players: UAVs; $U_{i}\;:i=1,\ldots ,N$;
2. A set of strategies for each player: This represents the next movement of the UAV in the 3D location at time (t), where *t* is defined as t=1,…,T. The selected strategy can be represented for each player $U_{i}$ at *t* by the location: $l_{U_{i}}\left(t\right)={\left[x_{U_{i}}\left(t\right),\;y_{U_{i}}\left(t\right),\;h_{U_{i}}\left(t\right)\right]}^{T}$. The UAV has the ability to move at multiple levels, and each level is limited by the min and max height; they can move in a set of directions. We constructed our list by 27 movements in the 3D space (i.e., UAV can move forward, backward, right, left, and diagonally, hover in the same location, and move up/down with the same previous options);
3. Utility/payoff: This depends on the coverage score $\left({IP}_{i}\left(t\right)\right)$ and the energy consumption $\left(E_{i}\left(t\right)\right)$.

The utility function depends on the energy consumption and the coverage score, and it can be designed as follows: UAVs consume energy for their sensing and movement processes. We first considered the energy consumption of the UAVs in the sensing process to cover the IPs in the candidate region. Each UAV has the ability to sense the downward area based on its own camera specifications. However, when the coverage radius increases, the UAV needs more energy to cover more area either by moving or by increasing the used power. We assumed that all UAVs had the same range of the sensing area due to the same physical properties and altitude limitations. To reduce the energy usage, the sensing range should be decreased, and it can be represented as a circular area with $R_{i}^{sens}=2\times r_{max}$ for all UAVs (i=1,2,…,N). Based on that, there is a tradeoff between the covered area and the energy usage, and it can be expressed mathematically based on the selected action $\left(a_{i}\left(t\right)\right)$ of the UAV as follows:

(14) $$E_{i}^{sens}\left(a_{i}\left(t\right)\right)=\beta_{s}{\left(a_{i}^{r}\left(t\right)\right)}^{2},$$

where $\beta_{s}$ is a constant depending on the efficiency of the sensing units of the UAV and $a_{i}^{r}\left(t\right)$ represents the normalized sensing radius based on the selected action (i.e., new location) at time (t), and each UAV attempts to find its own energy usage by finding a suitable radius.

Next, we considered the energy consumption resulting from the movements of the UAVs, and this value depends on both the current and previous locations of the UAV. It can be represented mathematically as follows:

(15) $$E_{i}^{mov}\left(a_{i}\left(t\right),a_{i}\left(t-1\right)\right)=\beta_{p}E_{\mathrm{cons}}\left(a_{i}\left(t\right),a_{i}\left(t-1\right)\right),$$

where $\beta_{p}$ is a constant depending on the efficiency of the power units for the UAV, $a_{i}\left(t\right)$ is the action for UAV (i) and represents the new location of the UAV $\left(l_{U_{i}}\left(t\right)\right)$, and $a_{i}\left(t-1\right)$ represents the previous location of the UAV $\left(l_{U_{i}}\left(t-1\right)\right)$.

Using the coverage score, fairness, sensing energy, and movement energy equations, the utility to be designed next is a function of the previous and current locations of the UAV. Since the control problem depends on the current state and the previous one, it can be implemented using the state-based potential game. The relation between these variables can be represented in a linear form or a non-linear form by adding some other weights and constants that describe the environment. To formulate the coverage–energy problem as a state-based game, the utility function $\left(U_{i}\right)$ was constructed for each UAV (i) to study the tradeoffs between the covered area and the energy consumption by UAV (i). Initially, the utility function for UAV (i) can be expressed as follows:

(16) $$U_{i}\left(a_{i}\left(t\right),a_{i}\left(t-1\right)\right)=F\left(a_{i}\left(t\right)\right)-E_{i}^{sens}\left(a_{i}\left(t\right)\right)-E_{i}^{mov}\left(a_{i}\left(t\right),a_{i}\left(t-1\right)\right),$$

where *F* is the coverage function for UAV *i*, which depends on the coverage score and the fairness, and it is expressed as $F\left(a_{i}\left(t\right)\right)=FI\left(a_{i}\left(t\right)\right)\times {IP}_{i}\left(a_{i}\left(t\right)\right)$. Note that $U_{i}$ is local over the covered area by UAV (i), and it is dependent only on the actions of UAV (i). As we noticed from the utility function in Equation (16), increasing energy consumption will have a negative impact on the utility value of the UAV. On the other hand, increasing the coverage score will have a positive impact on the utility value while taking into considerations the connectivity and the interference issues between UAVs, as well as the borders of the candidate region and the minimum distances between UAVs. In this problem, the main objective is to maximize the utility value for each UAV, and hence, we looked for the actions that satisfy:

(17) $$A^{*}=\arg\max_{a\in A}U\left(a\left(t\right)\right)\;.$$

After introducing the game ingredients and based on [55], the state-based potential game is discussed in the following definition:

**Definition** **1.** *The coverage–energy-state-based potential game G:=(N,A,U), where $U=\left\{U_{i},i=1,\ldots ,N\right\}$ and A is the action set, is an exact state-based game with the following potential function:*

(18) $$\Phi_{1}\left(a\left(t\right),a\left(t-1\right)\right)=\sum_{i=1}^{N}\left(F_{i}\left(a\left(t\right)\right)-E_{i}^{sens}\left(a\left(t\right)\right)-E_{i}^{mov}\left(a\left(t\right),a\left(t-1\right)\right)\right).$$

The proof part of the above equation is in Appendix A. Algorithm 1 summarizes the main steps of the state-based game part.
**Algorithm 1:** Pseudocode for the state-based game part.1.21:Initialize the UAV network.2:**for** each timeslot *t* in *T* **do**3:    **for** each UAV *i* in *N* **do**4:        **for** each action *j* in actions **do**5:           Evaluate IP and FI values using action *j*.6:           Find $E_{sens}$ and $E_{mov}$ using action *j*.7:           Store $IP,FI,E_{sens},E_{mov}$.8:        **end for**9:        Select the action with the maximum reward value using Equation (16).10:        Update the UAV location based on the action.11:        **while** The new UAV location is outside the region or the UAV loses its connectivity **do**12:           Cancel the new movement.13:           Select the next maximum action.14:           Update the UAV location based on the new action.15:        **end while**16:        Update the last $IP,FI,E_{sens},E_{mov}$ values for UAV *i*.17:    **end for**18:**end for**

When the UAV changes its action, the utility will not be necessarily improved with other UAVs’ actions. Hence, it does not definitely mean that the action is a global optimum for all the network. Due to this and the sudden changes in the network, the UAV network should have some learning capabilities to avoid such cases.

### 4.2. Learning Approach

The UAV network should have online learning capabilities especially in the case of unknown environments or sudden changes in the candidate region during the mission. Moreover, with the increasing complexity of the networks, most of the proposed game approaches are unable to achieve the requirements and reach a stable point. Hence, a learning approach is required to overcome such behaviors that can occur in the UAV networks. One of the popular learning approaches is reinforcement learning (RL); hence, we start by reviewing RL and then introduce the proposed learning approach.

#### 4.2.1. Preliminaries and Problem Model

The deep RL (DRL) approach considers the “deep” shape of RL, which consists of two phases: training phase and testing phase. In the training phase, the DNN is trained offline, and the exploration stage is required to find the optimal policy. In the testing phase, it consumes less resource, and there is no need for the exploration stage compared to the training phase; it only performs forward propagation. DRL within the actor–critic framework [56] consists of the critic $Q(s,a\left|\theta^{Q}\right.)$, which finds the action value function using the actor policy $\pi (s\left|\theta^{\pi }\right.)$, where $\theta^{Q}$ and $\theta^{\pi }$ are the parameters of the critic and actor networks, respectively.

Two methods are usually used to overcome the divergence problem that results from using the DNN in DRL: the target network and the experience replay buffer [24]. The DRL approach extracts and samples a mini-batch of the collected experiences during the training from the replay buffer. The generated random samples break the relation between the sequential samples and make the training process more stable. The target networks of the critic and actor, $Q^{{}^{\prime }}\left(s,a|\theta^{Q^{{}^{\prime }}}.\right)$ and $\pi^{{}^{\prime }}\left(s|\theta^{\pi^{{}^{\prime }}}.\right)$ have the same configurations of the learned network $\left(Q\left(s,a\left|\theta^{Q}\right.\right),\pi \left(s_{t}\left|\theta^{\pi }\right.\right)\right)$ and are employed to evaluate the update target.

In the UAV network, a UAV is treated as an agent and interacts with the environment in discrete decision epochs/timeslots. At each timeslot *t*, the UAV i observes the state $s_{t}$, which enters the network as the input, and it outputs the action $a_{t}$. Next, the UAV receives a reward value $r_{t}$, and the state converts to $s_{t+1}$. We need to find a policy $\pi (s_{t}\left|\theta^{\pi }\right.)\;$ that converts a state into an action to improve the discounted cumulative reward $R_{0}$ = $\sum_{t=0}^{T}\gamma r(s_{t},a_{t})$, where γ∈[0,1] represents the discount factor. A replay buffer is used to store the experience values $\left(s_{t},a_{t},\;r_{t},\;s_{t+1}\right)$ for the training of the network. In the case of state $s_{t}$, the system follows action $a_{t}$ at epoch *t*, then:

(19) $$Q\left(s_{t},a_{t}\right)=E\left[R_{t}\left|s_{t},a_{t}\right.\right],$$

where $R_{t}=\sum_{j=t}^{T}\gamma r(s_{j},a_{j})$, and it represents the discounted cumulative reward; $Q\left(s_{t},a_{t}\right)$ estimates the expected value of $R_{t}$ for each $\left(s_{t},a_{t}\right)$ pair. The greedy policy is one of the commonly used off-policies, where $\pi \left(s_{t}\left|\theta^{\pi }\right.\right)=\arg\max_{a_{t}}Q\left(s_{t},a_{t}\right)\;$. The critic network is trained by minimizing the following loss function:

(20) $$L\left(\theta^{Q}\right)=\frac{1}{L}\sum_{b=1}^{L}{\left[y_{t}\left(b\right)-Q\left(s\left(b\right),a\left(b\right)\left|{\;\theta }^{Q}\right.\right)\right]}^{2},$$

(21) $$y_{t}\left(b\right)=r_{t}\left(b\right)+\gamma Q^{{}^{\prime }}\left(s\left(b+1\right),\pi^{{}^{\prime }}\left(s\left(b+1\right)\left|{\;\theta }^{\pi^{{}^{\prime }}}\right.\right)\left|{\;\theta }^{Q^{{}^{\prime }}}\right.\right),$$

where *L* is the mini-batch size from the reply buffer B. To train the actor network, we need to minimize the following loss function of the actor:

(22) $$L\left(\theta^{\pi }\right)=\frac{1}{L}\sum_{b=1}^{L}-Q\left(s\left(b\right),\pi \left(s\left(b\right)\left|{\;\theta }^{\pi }\right.\right)\left|{\;\theta }^{Q}\right.\right),$$

The parameters of the target networks $\left(\theta^{Q^{{}^{\prime }}},\theta^{\pi^{{}^{\prime }}}\right)$ are updated using the following expressions as explained before and the use of the gradient method [28]:

(23) $$\theta^{Q^{{}^{\prime }}}=\varepsilon \theta^{Q}+\left(1-\varepsilon \right)\theta^{Q^{{}^{\prime }}},$$

(24) $$\theta^{\pi^{{}^{\prime }}}=\varepsilon \theta^{\pi }+\left(1-\varepsilon \right)\theta^{\pi^{{}^{\prime }}},$$

where ε represents a constant to control the soft update [57].

#### 4.2.2. State Space

The UAV *i* at timeslot *t* in the system acts a control center, which adjusts its location and power transmission. The observation space $O_{t}^{i}$ of the coverage–energy problem contains: UAV locations $\left(x_{t}^{i},y_{t}^{i},h_{t}^{i}\right)$ and the energy consumption $e_{t}^{i}$ of all UAVs. Specifically, $O_{t}^{i}$ can be represented as follows:

(25) $$O_{t}^{i}={\left\{x_{t}^{i},y_{t}^{i},h_{t}^{i},e_{t}^{i}\right\}}_{\left(i\in N,\;\;t=1,2,\ldots ,T\right)}$$

Based on the observation space, the state space of the system at timeslot *t* for any UAV *i* can be written using the coverage score. Specifically, it can be represented as follows:

(26) $$s_{t}^{i}={\left\{x_{t}^{i},y_{t}^{i},h_{t}^{i},e_{t}^{i}\right\}}_{\left(i\in N,\;\;t=1,2,\ldots ,T\right)}$$

$s_{t}^{i}$ has a cardinality of (4N), and the DRL agent makes decisions based on both the energy consumption and the coverage score (i.e., location).

#### 4.2.3. Action Space

Each UAV needs to select the most appropriate next location with the minimum energy consumption during its flight period. The action $a_{t}^{i}$ of UAV *i* at timeslot *t* is the next location and can be represented as follows:

(27) $$a_{t}^{i}={\left\{x_{t}^{i},y_{t}^{i},h_{t}^{i}\right\}}_{\left(i\in N,\;\;t=1,2,\ldots ,T\right)}$$

$a_{t}^{i}$ has a cardinality of (3N), and it is defined as a control policy that defines how the UAV moves at each decision timeslot.

#### 4.2.4. Reward Function

As we explained before, the UAV can move in restricted movements, where each UAV should not cross the borders of the candidate region and should not move close to other UAVs in the network. Therefore, we added a fine $\left(f_{t}^{i}\right)$ to the UAV that crosses the border. Moreover, the UAV will lose its connections with other UAVs based on its communication range $R_{c}$. This fine value forces the UAVs to avoid selecting the actions that lead them to move outside the region and lose the connections with other UAVs. The network efficiency at timeslot *t* can be defined as follows:

(28) $$U_{t}=F_{t}-\left(E_{t}^{sens}+E_{t}^{mov}\right)$$

The first term of the reward is the gain (coverage), while the second term is the cost (energy consumption). The reward function is then expressed mathematically for UAV *i* at timeslot *t* as follows:

(29) $$r_{t}^{i}={\left\{U_{t}-f_{t}^{i}\right\}}_{\left(i\in N,\;\;t=1,2,\ldots ,T\right)}$$

#### 4.2.5. Training Process

The coverage–energy problem algorithm was designed to be an episode from the start of the UAVs’ flight from the initial locations to the end of the energy consumption. Algorithm 2 illustrates the learning process for the UAV based on the previous specifications. Due to the huge state space and action space and in order to remove the redundancy and improve the accuracy of the simulation, we used offline and online learning to train the network. Each UAV has unique actor and critic networks, and the target network of the UAV is a copy from this actor and critic networks. However, the weights of the target network are updated separately using Equations (23) and (24). As explained before, the algorithm learns from the experiences (i.e., action, state, and reward) that are stored in the replay buffer with a size of (B). In other words, at each timeslot *t* during the learning process, the actors and critics for all UAVs in the network are updated from the experiences with the use of randomly sampled mini-batch (L).
**Algorithm 2:** Pseudocode for the DRL approach.1.21:Initialize the experience replay buffer B.2:**for** each UAV *i* in *N* **do**3:    Initialize the actor network $\pi \left(s_{t}\left|\theta^{\pi }\right.\right)$ with weights $\theta^{\pi }$.4:    Initialize the critic network $Q\left(s,a\left|\theta^{Q}\right.\right)$.5:    Initialize the target actor network $\pi^{{}^{\prime }}\left(s\left|\theta^{\pi^{{}^{\prime }}}\right.\right)$ with weights $\theta^{\pi^{{}^{\prime }}}$.6:    Initialize the target critic network $Q^{{}^{\prime }}\left(s,a\left|\theta^{Q^{{}^{\prime }}}\right.\right)$ with weights $\theta^{Q^{{}^{\prime }}}$.7:**end for**8:**for** each episode in H **do**9:    Initialize the locations of the UAVs.10:    The initial speed is zero for the UAVs, and their battery energy is $E_{max}$.11:    Initialize the environment.12:    Receive the initial state $s_{1}$.13:    **for** each time *t* in *T* **do**14:        **for** each UAV *i* in *N* **do**15:           Select action $a_{t}^{i}=\pi^{i}\left(s_{t}\left|\theta^{\pi }\right.\right)+N$, where N is the noise term.16:        **end for**17:        UAVs execute their actions $a_{t}=\left(a_{t}^{1},\ldots ,a_{t}^{N}\right)$.18:        Update next state $s_{t+1}$, and obtain reward $r_{t}=\left(r_{t}^{1},\ldots ,r_{t}^{N}\right)$.19:        **for** each UAV *i* in *N* **do**20:           **if** UAV *i* moves outside the region or close to other UAVs **then**21:               Find $r_{t}^{i}=U_{t}-f_{t}^{i}$.22:               Neglect the new location and update $O_{t}^{i}$.23:           **end if**24:        **end for**25:        Update $s_{t}⟵s_{t+1}$.26:        Store $\left(s_{t},a_{t},r_{t},s_{t+1}\right)$ in the buffer.27:        **for** each UAV *i* in *N* **do**28:           Sample *L* random mini-batches $(s_{t},a_{t},r_{t},s_{t+1})\in B$.29:           Find $y_{t}\left(b\right)=r_{t}\left(b\right)+\gamma Q^{{}^{\prime }}\left(s\left(b+1\right),\pi^{{}^{\prime }}\left(s\left(b+1\right)|{\;\theta }^{\pi^{{}^{\prime }}}.\right)|{\;\theta }^{Q^{{}^{\prime }}}.\right)$, where b=1,…,L.30:           Update weights $\theta^{Q}$ by minimizing: $L\left(\theta^{Q}\right)=\frac{1}{L}\sum_{b=1}^{L}{\left[y_{t}\left(b\right)-Q\left(s\left(b\right),a\left(b\right)\left|{\;\theta }^{Q}\right.\right)\right]}^{2}$.31:           Update weights $\theta^{\pi }$ by minimizing: $L\left(\theta^{\pi }\right)=\frac{1}{L}\sum_{b=1}^{L}-Q\left(s\left(b\right),\pi \left(s\left(b\right)\left|{\;\theta }^{\pi }\right.\right)\left|{\;\theta }^{Q}\right.\right)$.32:           Update the target network’s weights: $\theta^{Q^{{}^{\prime }}}=\varepsilon \theta^{Q}+\left(1-\varepsilon \right)\theta^{Q^{{}^{\prime }}}$ and $\theta^{\pi^{{}^{\prime }}}=\varepsilon \theta^{\pi }+\left(1-\varepsilon \right)\theta^{\pi^{{}^{\prime }}}$.33:        **end for**34:    **end for**35:**end for**

From Algorithm 2, we can see the pseudocode of the training process for the learning approach. It starts by initializing the replay buffer, and then, each UAV randomly initializes its actor and critic networks with weights $\theta^{\pi }$ and $\theta^{Q}$, respectively. Furthermore, the weights of the target network ${(\theta^{\pi^{{}^{\prime }}},\theta }^{Q^{{}^{\prime }}})$ are randomly initialized for all UAVs in the same manner as the actor and critic networks. Next, the training is configured by having H episodes, and each episode consists of *T* timeslots. In the training loop, the system obtains initial state $s_{1}$, and we construct the starting conditions of the environment. For each UAV *i*, it selects an action $a_{t}$ according to the actor $\pi^{i}\left(s_{t}\left|\theta^{\pi }\right.\right)$ with the observation $O_{t}^{i}$ as the inputs. To avoid the UAV selecting a locally optimal policy and performing more explorations, a noise term with a Gaussian distribution is added to the selected action. After performing these actions, the UAV will obtain a reward value $r_{t}$ and a new state $s_{t+1}$. However, a fine is applied on the UAV, if the selected action forces the UAV to go outside the region or close to other UAVs. Hence, the UAV will avoid this action, and it cancels the new location. The final values of $\left(s_{t},a_{t},r_{t},s_{t+1}\right)$ are then stored in the replay buffer. At the end of the training process, each UAV at timeslot *t* randomly selects a mini-batch with a length of (L) samples from the buffer, and it then evaluates the target value $\left(y_{t}\right)$ using the target critic $Q^{{}^{\prime }}\left(s,a\left|\theta^{Q^{{}^{\prime }}}\right.\right)$. After this, the weights of the critic and actor network $\left(\theta^{Q},\theta^{\pi }\right)$ are updated using the loss $L\left(\theta^{Q}\right)$ and the gradient method, respectively. Lastly, the target network weights ${(\theta }^{Q^{{}^{\prime }}},\theta^{\pi^{{}^{\prime }}})$ are updated slowly using $\left(\theta^{Q},\theta^{\pi }\right)$ and the learning rate ε.

#### 4.2.6. Complexity Analysis

In dynamic scenarios, we cannot determine the trajectory of the UAVs due to their unpredictable movements. Thus, the time complexity analysis of the UAV control algorithm is very important. With the increasing complexity of the UAV control problems, the basic learning approach is not suitable to meet the requirements of the distributed algorithm in dynamic scenarios. In our research, the control problem depended on many factors, the most important factors being the energy and the location of the UAVs. Therefore, a learning algorithm was needed to solve the control problem during the UAV movements, which was a combination of the state-based game and the actor–critic algorithm.

As for the complexity analysis of the SBG-AC algorithm, the complexity of the exploration and learning process depended on both the number of states and the number of actions in the control problem, as well as the architecture of the learning model. Due to the huge space and action spaces, a DNN was used in the learning process for both the actor and critic networks. In the SBG-AC algorithm, the UAV chooses its action based on the current state in each timeslot to construct the training dataset. With enough training steps for a network with a number of UAVs, a huge number of training samples were collected, which guaranteed the convergence of the DNN and enabled the UAV to learn the optimal action. Since SBG-AC can be learned offline, we considered the time complexity of the testing stage. In the testing stage, the collected observations were the input of the DNN, and the selected action was the output. Hence, the complexity for the DNN with fully connected layers depended on the number of neurons and the number of layers in the network and can be expressed mathematically as follows:

(30) $$Complexity=O\left(\sum_{l=1}^{N_{layers}}N_{l}\times N_{\left(l-1\right)}\right),$$

where $N_{l}$ represents the number of neurons in the fully connected layer (l) and $N_{layers}$ is the number of fully connected layers in the model.

## 5. Performance Evaluation

In this section, we first present the simulation setting and then evaluate and discuss the results for the game and learning model. To highlight the performance of the SBG-AC, we considered a set of experiments with different numbers of UAVs and initial settings. In addition, we simulated another two models from previous research for the performance comparisons [31,36].

### 5.1. Simulation Settings

In our simulation, we performed the simulation runs with Tensorflow 2.0.0 and Python 3.7, and other specifications of the machine are listed in Table 2. Due to random initialization in the UAVs’ locations, we repeated our simulation scenarios 100 times, and we found the average values of the targeted metrics. We considered a square area with 100 × 100 square units; the center of each unit is called the IP, and its side length equaled 100 m. In addition, when the UAV covers part of the square or the whole square, it considers it as a covered square based on the probability function described in Equation (9). UAV (u) can fly in 3D space at time *t* with $\left(x_{u}\left(t\right),\;y_{u}\left(t\right),\;h_{u}\left(t\right)\right)$ coordinates. To avoid collisions and keep UAVs connected most of the time, we restricted the movements of the UAVs based on Equations (1) and (2), and we penalized the UAV when it lost its connections or moved outside the area by one. Furthermore, the UAVs could move vertically within a predefined range of altitude values due to UAV regulations and rules. The fully charged energy of the battery was $E_{max}=1\times 10^{5}$ joules. The channel characteristics of the UAV network followed the urban environment with path-loss exponent $\left(n_{0}=2.5\right)$, and more settings of the network are summarized in Table 3 based on [31,36,40,44,45].

### 5.2. Network Architecture

**Figure 2:**
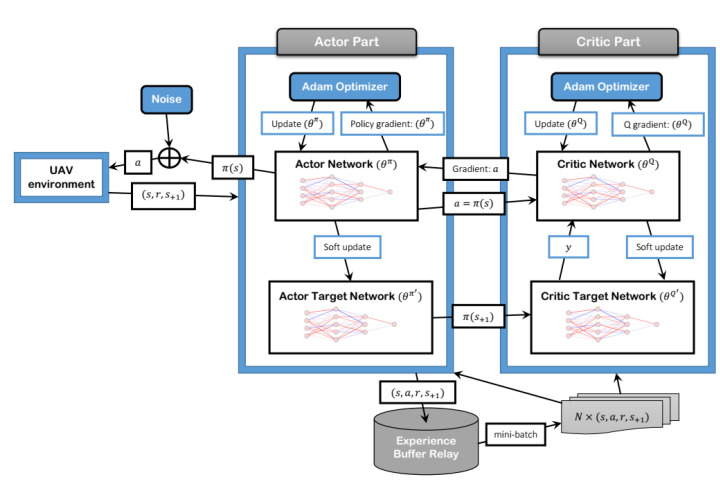
Actor–critic network.

The learning process of the SBG-AC model was designed based on the actor–critic DRL algorithm and the deep deterministic policy gradient (DDPG). The actor–critic network is shown in Figure 2. Both actor and critic networks were developed using DNNs. Each network consisted of a set of hidden layers with a predefined number of neurons. The actor and critic DNNs need to have a large size to handle the learning data and prevent the over-fitting problem. Hence, we conducted a set of experiments using “Tensorflow 2.0.0” to find the optimal values of the hyperparameters for the actor–critic DNNs. Both networks had two fully connected hidden layers. The neurons of the actor network were set to 1000 in the first hidden layer and 500 in the second hidden layer. On the other hand, the neurons in the critic network were set to 500 and 400 neurons in the first and second hidden layers, respectively. The input and output sizes of the two networks depended on the action and state sizes and the number of UAVs in the model.

In our design, the actions represent the next 3D locations of the UAVs with a size equal to (3×N), and the states represent the 3D location and the residual energy value for each UAV with a size equal to ((3+1)×N). To improve the learning model and avoid convergence instability or local optima, we normalized the states of the UAV to [0,1] and used the scaling and tanh methods to bound the actions in the actor network to [−1,1]. Moreover, we used the rectified linear unit (ReLU) function for activation purposes in all layers except the output layer and L2 regularization for over-fitting prevention in both networks. The Adaptive moment estimation (Adam) optimizer [58] was used in the actor and critic networks to update the DNNs with learning rates equal to 0.001 and 0.002, respectively. We used (ε) equal to 0.01 in the soft updating process, and the discount value (γ) was set to 0.999. Most of the settings in the two networks were used after performing some trial experiments. All settings for the learning model are listed in Table 4.

During the training phase, we stored the trained SBG-AC results every fifty episodes, each of which had two-hundred epochs, and thus, we had eight models in total. In the testing phase, we tested each model 10 times. We then found the average value and selected the best one from the eight models.

### 5.3. Evaluation Metrics

In this paper, we used three metrics to evaluate the performance of the model, and we plotted the normalized values of the selected metrics for different numbers of UAVs. The three selected metrics were:

1. Average coverage score: This was evaluated using the coverage probability Equation (9). In each iteration, the coverage score was updated based on the new movement of the UAVs, where the new location for the UAV was selected based on the resulting action, which improved the reward value for the UAV network. With any network size, we found the coverage probability for all cells in the selected area over the running period;
2. Fairness index: This shows how the UAVs cover the ground points (IPs) in the network. To have a fair model, we needed to avoid covering some cells all the time and rarely covered other cells. The coverage performed by the UAVs should be equally distributed on all cells to achieve the best fairness value. This metric was measured using Equation (10);
3. Normalized energy consumption: This represents the required energy for sensing and movement processes during the testing period. We recorded the energy consumption for each UAV and then found the average energy consumption for all UAVs in the network within the current iteration/timeslot. Next, we normalized the measured values to the required energy for the UAV to move the maximum distance. The sensing and movement consumption energy value was evaluated using Equation (12).

### 5.4. Benchmark Models

To compare and validate the SBG-AC model using the same simulation settings, we simulated the following learning models:

1. DRL-EC3 [31]: This is a learning method that deploys and navigates the UAVs to improve the energy efficiency of the UAV network. This model was built with the help of DRL technology, and all UAVs worked based on one actor–critic network;
2. Distributed-DRL [36]: This is an enhanced version of the DRL-EC3 model. It was built to handle the dynamic changes in the network, let the UAVs work in a distributed manner, and ensure the connectivity among all UAVs. A learning-based method was developed, where each UAV had its own actor–critic network, and it was able to decide its best action.

The similarities and differences among SBG-AC, DRL-EC3, and distributed-DRL are summarized in Table 5.

### 5.5. Performance and Analysis

**Figure 3:**
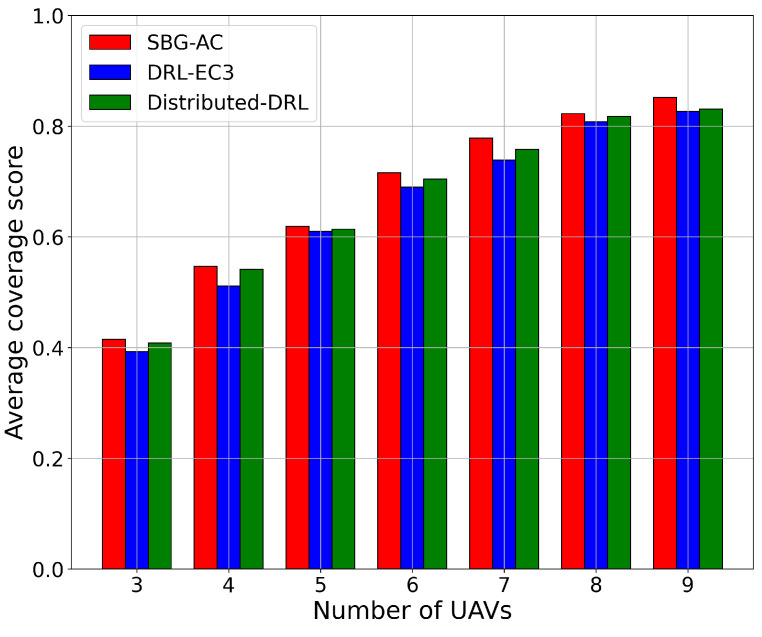
Average coverage score value for a 3-, 4-, 5-, 6-, 7-, 8-, and 9-UAV network.

We started by comparing the performance of the three models in terms of the coverage score with respect to the number of UAVs. Figure 3 demonstrates the results in terms of the coverage score for SBG-AC (red color), DRL-EC3 (blue color), and distributed-DRL (green color) for different numbers of UAVs. From Figure 3, we noticed that SBG-AC beat DRL-EC3 and distributed-DRL in terms of the coverage score by an average increment approximately equal to 3% and 1.1%, respectively, for all network sizes. For instance, when the number of UAVs = 3, SBG-AC achieved a coverage score equal to 42%, while DRL-EC3 covered around 39.5% and around 40.8% with distributed-DRL for the same area. In the case of seven UAVs, the coverage score achieved by SBG-AC was 78%, by DRL-EC3 was 74.2%, and by distributed-DRL was 73.8% for the same network. Furthermore, in the case of nine UAVs, SBG-AC outperformed DRL-EC3 and distributed-DRL by around 2% and 1% with coverage scores equal to 85.2%, 84%, and 83.1%, respectively. The same trend was achieved by the three models for other sizes. When the number of UAV increased, the average coverage score reached by SBG-AC monotonically improved due to the new UAVs being able to have more flexibility in covering IPs, thus achieving a better coverage score. The initial locations for the UAVs in these models were assigned using a uniform random distribution. We also noticed that the average needed epochs to reach the same steady-state coverage value were fewer for SBG-AC compared to DRL-EC3 and distributed-DRL in all scenarios.

**Figure 4:**
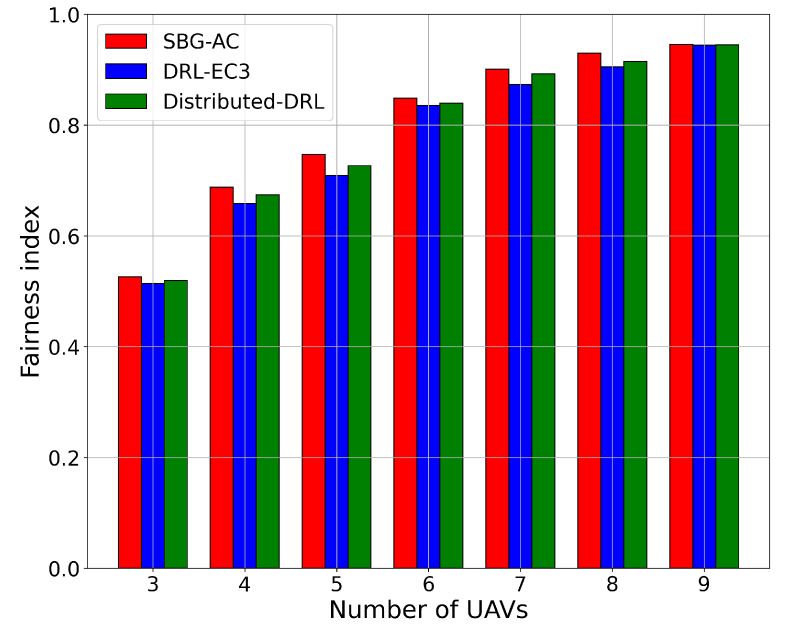
Fairness index for a 3-, 4-, 5-, 6-, 7-, 8-, and 9-UAV network.

Next, we compared the three models in terms of the fairness index with respect to the number of UAVs in the network. Figure 4 presents the results in terms of the fairness index for SBG-AC (red color), DRL-EC3 (blue color), and distributed-DRL (green color) for different numbers of UAVs. From Figure 4, we noticed that SBG-AC achieved better fairness values in all networks compared to DRL-EC3 and distributed-DRL by an average increment approximately equal to 2.2% and 1.1%, respectively. For instance, when the number of UAVs was four, SBG-AC reached a fairness index equal to 69%, while DRL-EC3 achieved a fairness index equal to 66% and distributed-DRL a fairness index equal to 67.8%. In the case of five UAVs, SBG-AC significantly improved the fairness index with a value equal to 74.7%, 70.5% for DRL-EC3, and 72.7% for distributed-DRL using the same network. In case of eight UAVs, SBG-AC outperformed DRL-EC3 and distributed-DRL by around 2.4% and 1.1%, with fairness values equal to 92.9%, 90.5%, and 91.4%, respectively. The same trend was seen for these models for other network sizes. When the number of UAVs increased in the network, the fairness index directly improved due to more cells being covered by the new UAVs. We also noticed that the average needed epochs to reach the same fairness index were fewer in SBG-AC compared to DRL-EC3 and distributed-DRL in all scenarios.

**Figure 5:**
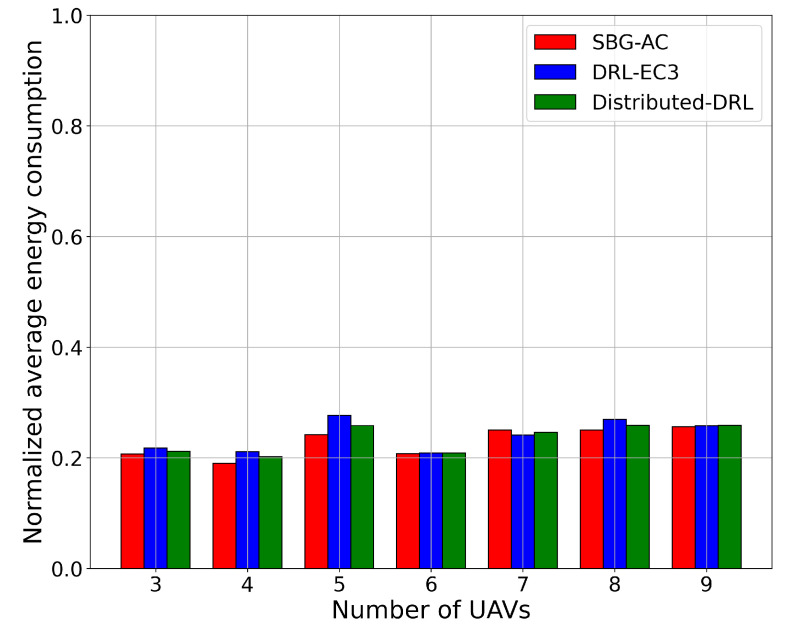
Normalized average energy consumption for a 3-, 4-, 5-, 6-, 7-, 8-, and 9-UAV network.

For the energy consumption metric, we compared the SBG-AC model with the two models in terms of the normalized average energy consumption for different numbers of UAVs. Figure 5 shows the normalized average energy consumption results for SBG-AC (red color), DRL-EC3 (blue color), and distributed-DRL (green color). From Figure 5, we noticed that SBG-AC, DRL-EC3, and distributed-DRL almost has the same energy consumption values for all network sizes. For instance, when the number of UAVs was six and nine, SBG-AC, DRL-EC3, and distributed-DRL required almost the same normalized energy consumption (20.8% and 25.7%) to reach a coverage of 71.5% and 85.2% in SBG-AC, 69.4% and 84% in DRL-EC3, and 70.4% and 83.1% in distributed-DRL. In the case of seven UAVs, DRL-EC3 required less normalized energy consumption (24%) compared to SBG-AC (25%), with coverage scores of 74.2% for DRL-EC3 and 78% for SBG-AC. For other sizes, SBG-AC beat DRL-EC3 and distributed-DRL with a small deviation in their values. We observed that the energy consumption values did not vary when the number of UAVs increased in these models. Indeed, more UAVs might lead to fewer movements compared to fewer UAVs due to the restrictions in the connectivity and interferences with other UAVs. As a result, the UAVs did not consume more energy for the movements.

### 5.6. Discussions

In this testing scenario, we used eight UAVs, and the noise term was zero variance, while other simulation settings were kept as before. Here, we discuss if the SBG-AC model has a practical sense to be applied for UAV control challenges such as energy, coverage, and fairness. Then, we compared it with DRL-EC3 and distributed-DRL models in terms of the three metrics.

Our reward function depends on three objectives (i.e., coverage, fairness, and energy), and it has a penalty value to keep the UAVs in the candidate area and to maintain the connectivity and interference among the UAVs. In the testing phase, the state data were the input (i.e., location and energy) of the system, and each UAV utilized its own actor network in a distributed manner to produce an action. The UAV selected its actions (i.e., next location) based on the designed reward function and the utilization of the actor network. However, when the UAV finds that the new location, it might move out of the area. Hence, the UAV will cancel the new movement, and it update its states accordingly.

From the summarized testing data in Table 6, we noticed that SBG-AC outperformed DRL-EC3 and distributed-DRL in the coverage and fairness metrics. In terms of coverage score per episode, with eight UAVs, SBG-AC covered around 84.6% of the candidate area, DRL-EC3 80.8%, and distributed-DRL 82.7%. For the fairness index, SBG-AC also achieved a better fairness ratio compared to DRL-EC3 with the improvement ratio equal to 3% for the same UAV network and 1.7% with distributed-DRL. In terms of energy consumption, the three models had almost the same normalized value, and hence, this indicated that the energy had less impact on the coverage and fairness values. As a result, these models provided more robustness to the network compared to the traditional models (i.e., random-based model). As indicated before, more movements introduced more energy consumption; hence, we restricted the UAV movements by following only the resulting action from the actor–critic network in order to save the energy and then increase the overall lifetime. Indeed, the actor–critic-based algorithm was used since it considered the action policies of all available agents in the network and it had the ability to learn the policies for control problems with a large state space. The complexity of the SBG-AC can be determined with respect to the action dimension, the state dimension, and the construction of the DNN in the actor–critic network, as explained before in the complexity analysis part.

**Figure 6:**
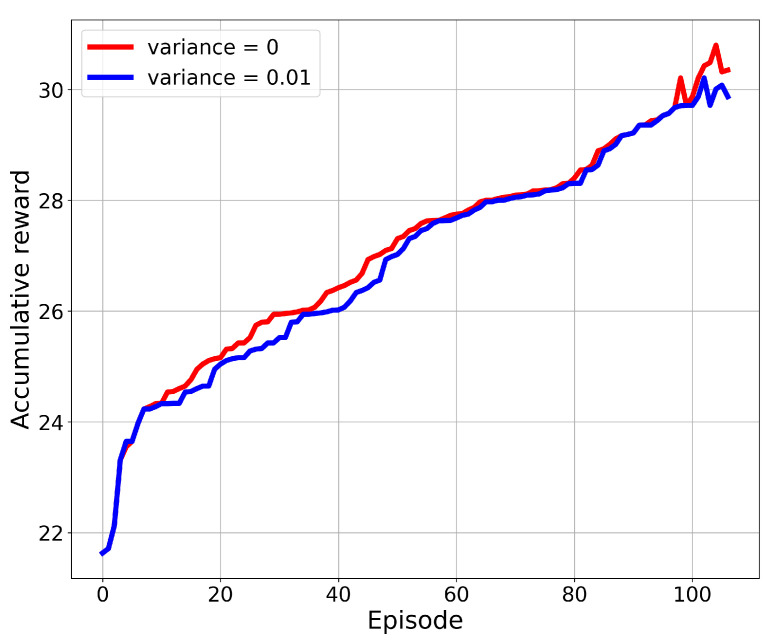
Accumulated reward over the number of episodes.

After the number of episodes in the training process, we found that the accumulative reward value for SBG-AC converged to a specific value without any improvement. Figure 6 presents the results in terms of achieved rewards over episodes for the eight-UAV network with zero variance noise and 0.01 variance noise. We noticed that the reward improved over the episode value due to the learning stage, then the growth in the reward after 50 episodes slowed down, and it started to converge. At the start of the simulation, many IPs still had not been visited by the UAVs, and the fairness index of the network was still low. With the learning process, an action will be selected based on the previous experience, and then, it will provide a significant improvement on the reward value until it converges. This convergence ensures that the model is designed in a proper way and it can work in different scenarios and under dynamic environments.

The summary and the advantages of using SBG-AC with eight UAVs are listed as follows:

1. SBG-AC improved the coverage score, fairness, and consumption energy by approximately 4%, 3%, and −0.6%, respectively, in the case of DRL-EC3 and by approximately 1.9%, 1.7%, and −0.4%, respectively, in the case of distributed-DRL;
2. SBG-AC needs fewer iterations/less time (=161) to achieve the best values of the three metrics compared to DRL-EC3 (=178) and distributed-DRL (=165).
3. Due to the use of instantaneous rewards and the action space (only the next locations), the performance of SBG-AC was higher than that of the DRL algorithm;
4. The dimensions of the input and output for the centralized algorithm increased with the number of UAVs; thus, the time complexity will increase accordingly. Hence, the distributed algorithm is more appropriate in the case of dynamic environments;
5. DNN was used in the actor and critic networks to estimate the state–action value instead of the using Q-table as in the basic RL models. Therefore, it is able to fit different functions based on different features, and the hyperparameters can be tuned (e.g., discount factor, neurons in the fully connected layer, and experience replay buffer size) to improve our results.

To this end, we used the actor–critic algorithm (i.e., DDPG), one of the most common DRL algorithms, in this research to take advantage of the above-mentioned results and features.

## 6. Conclusions and Future Work

In this paper, we introduced a novel algorithm called the state-based game with actor–critic (SBG-AC) to control multiple UAVs in order to improve the coverage, connectivity, and fairness and minimize the energy consumption in a UAV network. The control problem of the 3D movement for a group of UAVs under fairness, coverage, and energy constraints was formulated as a state-based potential game, while an actor–critic algorithm was proposed to solve the formulated game and to guarantee the convergence of the distributed model. In the SBG-AC model, each UAV acted as an agent in the system to search and learn its 3D location policy. We conducted extensive simulations for different numbers of UAVs (i.e., 3, 4, 5, 6, 7, 8, and 9) for the performance evaluation. We also found the proper settings of the actor–critic algorithm (i.e., DNNs’ configuration, learning rate, and discount factor) through a trial simulation. The simulation results demonstrated the efficiency and the convergence of SBG-AC, and it achieved better performance compared to DRL-EC3 and distributed-DRL in terms of fairness, coverage score, and energy consumption. These models converged to a final reward value in all network configurations, and this ensured the validity and the adaptability of the models. In future work, we will try to change the style of the action list and study the new behavior of the model. Furthermore, we will consider a variable velocity for the UAVs instead of a constant value.

## Figures and Tables

**Table 1:** Literature summary of game- and learning-based models.

Ref.	Method	Type	Objective(s)	2D/3D	Utility/Reward	UAVs	Metrics
[21]	Potential game	Game	Coverage maximization and power control	2D	Coverage and transmission power	4 to 9	Coverage with iterations, UAVs’ power
[32]	Potential game	Game	Maximize coverage	2D	Coverage probability	15	Coverage with iterations
[31]	DRL	Learning	Maximizes energy efficiency	2D	Coverage score, fairness index, and energy consumption	5 to 10	Coverage score, fairness index, energy consumption, and energy efficiency
[38]	Actor-Critic	Learning	Scheduling and power allocation	3D	Energy consumption	1	Energy
[40]	Q-learning	Learning	Coverage and capacity needs	3D	Energy consumption	1	Reward, energy, and coverage
[44]	DDPG	Learning	Enhance energy efficiency and allocate frequency band for fair communication	3D	Throughput fairness and energy consumption	1	Reward and speed

**Table 2:** Machine/software specifications.

Hardware/Software	Description
Processor	Intel(R) Xeon(R) Gold 5218 CPU @ 2.30 GHz 2.29 GHz
Operating System	Microsoft Windows 10 Professional x64
Memory	256 GB
Python	v3.7
Tensorflow	v2.0.0

**Table 3:** Simulation setting for the UAV network.

Parameter	Notation	Value	Parameter	Notation	Value
Number of UAVs	*N*	[3,4,5,6,7,8,9]	LoS attenuation	$\chi_{LoS}$	1 dB
Transmission power	*P*	32 dBm	NLOS attenuation	$\chi_{NLoS}$	20 dB
Cells/squares	*Q*	100×100	Environmental constants	C,B	0.11, 0.6
Timeslot	ΔT	1 s	Elevation angle	θ	45
Duration/iterations	*T*	200	LOS link	$k_{1},k_{2}$	10.39, 0.05
UAV speed	$v_{u}$	10 m/s	NLOS link	$g_{1},g_{2}$	29.06, 0.03
Path-loss exponent	$n_{0}$	2.5	Tip speed	$U_{tip}$	120 m/s
Carrier frequency	$f_{c}$	2 GHz	Mean rotor-induced velocity	$v_{0}$	0.002 m/s
Speed of light	*c*	0.3 Gm/s	Fuselage drag ratio	$d_{0}$	0.48
Air density	ρ	1.225 kg/m${}^{3}$	Rotor solidity	$s_{0}$	0.0001
disc rotor area	*A*	0.5 s${}^{2}$	Blade profile power	$P_{0}$	99.66 W
Sensing and power constants	$\beta_{s},\;\beta_{p}$	Random (0.8–1)	Derived power	$P_{1}$	120.16 W

**Table 4:** Parameters for the actor and critic networks.

Parameters	Actor	Critic
Number of hidden layers	2	2
Neurons per Hidden Layer 1	1000	500
Neurons per Hidden Layer 2	500	400
Activation function in hidden layers	ReLU	ReLU
Activation function in output layer	tanh	ReLU
Learning rate	0.001	0.002
Loss function	Equation (22)	Equation (20)
Optimizer	Adam	Adam
Batch size	64	
Memory capacity	5000	
Discount factor	0.999	
Noise variance	0 and 0.01	
Episode	400	

**Table 5:** Description of the SBG-AC, DRL-EC3, and distributed-DRL models.

Features	SBG-AC	DRL-EC3	Distributed-DRL
Simulation setting	Same setting in Table 3	Same setting in Table 3	Same setting in Table 3
Model technology	Game + learning	Learning	Learning
Altitude	Varied with a predefined range (3D)	Fixed for all UAVs (2D)	Fixed for all UAVs (2D)
Network restrictions	Boundaries and connectivity	Boundaries and connectivity	Boundaries and connectivity
State and action spaces	3D location	Horizontal flying angle and 2D distance	Horizontal flying angle and 2D distance
Reward function	Equation (28)	$\frac{Coverage\times Fairness}{EnergyConsumption}$	
Learning architecture	Multiple actor- critic networks	One actor- critic network	Multiple actor- critic networks
Network type	Distributed	Centralized	Distributed

**Table 6:** Performance comparison for an 8-UAV network.

Metrics	SBG-AC	DRL-EC3	Distributed-DRL
Coverage score per episode	0.846	0.808	0.827
Fairness index per episode	0.934	0.905	0.917
Normalized average energy consumption per episode	0.263	0.269	0.267

## Data Availability

Not applicable.

## Appendix A

**Proof.** The state-based potential game should achieve the following two conditions:

1. For any UAV i=1,…,N and action $a_{i}^{{}^{\prime }}\left(t\right)\in A_{i}$:
  (A1) $$\begin{array}{c} \Phi_{1}\left(a_{i}^{{}^{\prime }}\left(t\right),a_{-i}\left(t\right),a\left(t-1\right)\right)-\Phi_{1}\left(a\left(t\right),a\left(t-1\right)\right)=\\ U_{i}\left(a_{i}^{{}^{\prime }}\left(t\right),a_{-i}\left(t\right),a\left(t-1\right)\right)-U_{i}\left(a\left(t\right),a\left(t-1\right)\right), \end{array}$$
  where $a_{-i}$ is the actions of all UAVs other than UAV (i) and $a_{i}^{{}^{\prime }}$ represents the alternative action for UAV *i*;
2. For any state $a^{{}^{\prime }}\left(t-1\right)$ in the support of $\left(a\left(t\right),a\left(t-1\right)\right)$, the inequality $\Phi_{1}\left(a\left(t\right),a^{{}^{\prime }}\left(t-1\right)\right)$$\geq \;\Phi_{1}\left(a\left(t\right),a\left(t-1\right)\right)$ holds.

We first verified the first condition. According to the previous equations for the utility and potential functions, we have:

(A2) $$\begin{array}{c} \Phi_{1}\left(a_{i}^{{}^{\prime }}\left(t\right),a_{-i}\left(t\right),a\left(t-1\right)\right)-\Phi_{1}\left(a\left(t\right),a\left(t-1\right)\right)=\\ F_{i}\left(a_{i}^{{}^{\prime }}\left(t\right),a_{-i}\left(t\right)\right)+E_{i}^{sens}\left(a_{i}^{{}^{\prime }}\left(t\right)\right)+E_{i}^{mov}\left(a_{i}^{{}^{\prime }}\left(t\right),a_{i}\left(t-1\right)\right)-F_{i}\left(a\left(t\right)\right)-E_{i}^{sens}\left(a_{i}\left(t\right)\right)\\ -E_{i}^{mov}\left(a_{i}\left(t\right),a_{i}\left(t-1\right)\right)=U_{i}\left(a_{i}^{{}^{\prime }}\left(t\right),a_{-i}\left(t\right),a\left(t-1\right)\right)-U_{i}\left(a\left(t\right),a\left(t-1\right)\right). \end{array}$$

To verify the second condition, the fact that $a^{{}^{\prime }}\left(t-1\right)$ is in the support of $\left(a\left(t\right),a\left(t-1\right)\right)$ implies that $a^{{}^{\prime }}\left(t-1\right)=a\left(t\right)$. Hence, we have:

(A3) $$\begin{array}{c} \Phi_{1}\left(a\left(t\right),a^{{}^{\prime }}\left(t-1\right)\right)-\Phi_{1}\left(a\left(t\right),a\left(t-1\right)\right)=\\ -E_{i}^{mov}\left(a_{i}\left(t\right),a_{i}^{{}^{\prime }}\left(t-1\right)\right)+E_{i}^{mov}\left(a_{i}\left(t\right),a_{i}\left(t-1\right)\right)\geq 0 \end{array}$$

We applied the fact that $E_{i}^{mov}\left(a_{i}\left(t\right),a_{i}^{{}^{\prime }}\left(t-1\right)\right)=0$, and this satisfies the second condition. □
